# Supplementary material for: Measuring Knowledge of Healthcare Providers on Pediatric Palliative Care with an Online Questionnaire Based on the National Core Curriculum in Italy
Source: Healthcare (Basel). 2023 Jul 7;11(13):1971. doi: 10.3390/healthcare11131971 (PMC10341144; doi:10.3390/healthcare11131971)
Supplement: Supplementary file 1 [file healthcare-11-01971-s001.zip › healthcare-2486468-supplementary.pdf]

Measuring perceived, wished and actual knowledge of healthcare providers about pediatric palliative care with an online questionnaire in Italy

Supplementary files

Table of Contents

Table S1. Online questionnaire... 2
Table S2. Total scores by socio-demographic characteristics of respondents 7
Table S3. Proportion of scores ≥ 4 for items of Whished Knowledge scale by professional profile .. 8

Table S1. Online questionnaire

Rilevazione delle conoscenze in cure palliative pediatriche (CPP) e terapia del dolore (TD)
Sei un medico/psicologo/infermiere che lavora anche o solo in ambito pediatrico?
Rispondi al questionario anonimo che ci servirà a capire quali sono i bisogni formativi in merito alle cure palliative e alla terapia del dolore in pediatria.
Se vuoi ulteriori informazioni sul progetto di ricerca, clicca qui. (entrare alla voce "Ricerca" del menù "Attività").
Il sondaggio fa parte del progetto di ricerca: "Continuità e Complessità in Pediatria", promosso dal Burlo e realizzato in collaborazione con le Aziende Sanitarie del SSR ed il finanziamento della Regione Friuli-Venezia Giulia."(*)
Per approfondimenti, chiarimenti o dubbi, contatta:
Roberta Vecchi, Cell. 320 43 89 125 | Elisa Zanello, Cell. 320 43 89 142 | E-mail: ricerca.continuita@burlo.trieste.it
(*)Progetto "*Continuità delle cure per minori con complessità assistenziale: bisogni e percorsi in Friuli-Venezia Giulia*", finanziato con i contributi per la ricerca clinica, traslazionale, di base, epidemiologica e organizzativa, di cui all'articolo 15, comma 2, lettera b), della legge regionale 17/2014. Trasmissione integrazioni, CUP C93C17001400007
* campo obbligatorio

**Quesito**
**A. DATI SOCIO - ANAGRAFICI**
**A.1 Sei un uomo o una donna?***
○ M ○ F
**A.2 Qual è la tua età?***
○ <30 anni ○ 30-45 anni ○ 46-60 anni ○ >60 anni
**A.3 Qual è il tuo profilo professionale?***
○ medico ○ psicologo ○ infermiere ○ altro
**A.4 Se sei un medico, indicaci la tua specializzazione:**
○ oncologia ○ anestesia e rianimazione ○ pediatria ○ cure palliative ○ altro
**A.5 Se sei un medico e hai risposto altro, descrivi la tua specializzazione:**
**A.6 Se sei uno psicologo, inserisci la tua qualifica:**
○ psicologo ○ psicoterapeuta ○ altro
**A.7 Se sei uno psicologo e hai risposto altro, descrivi la tua qualifica (per es. studente in psicologia, tirocinante post laurea)::**
**A.8 Se sei un infermiere, inserisci la tua qualifica:**
○ infermiere pediatrico ○ infermiere professionale ○ altro
**A.9 Se sei un infermiere e hai risposto altro, descrivi la tua qualifica (per es.studente, referente infermierisico)::**

A.10 Indicaci la tua sede di lavoro principale (indica l'area):*

- ☐ Trieste
- ☐ Udine
- ☐ Pordenone
- ☐ Gorizia

A.11 Quanta parte della tua attività lavorativa riguarda pazienti pediatrici (0-17 anni)?*

○ 100% ○ >50% ○ <50% ○ 0%

A.12 Qual è il tuo contesto di lavoro?*

- ☐ ospedale
- ☐ territorio
- ☐ ente convenzionato
- ☐ ambulatorio MMG
- ☐ ambulatorio PLS

A.13 Descrivi l'Unità Operativa o il Servizio in cui lavori:

A.14 Qual è la tua anzianità di servizio?*

○ <5 anni ○ 5-10 anni ○ >10 anni

A.15 Qual è la tua formazione specifica in CP/CPP?

- ☐ master in CP
- ☐ master in CPP
- ☐ corsi di perfezionamento in CP
- ☐ corsi di perfezionamento in CPP
- ☐ congressi/seminari
- ☐ nessuna

A.16 Hai esperienza lavorativa in CP/CPP?*

○ solo CP ○ solo CPP ○ entrambe ○ nessuna delle due

B. QUANTO CONOSCI I SEGUENTI TEMI (esprimi la tua valutazione su una scala da 1 a 5, dove 1=per nulla, 2=poco, 3=abbastanza, 4=molto e 5=moltissimo)

B.1 Definizione e filosofia di TD e CPP*

○ 1 ○ 2 ○ 3 ○ 4 ○ 5

B.2 Diritti, normative, legislazione specifica in TD e CPP*

○ 1 ○ 2 ○ 3 ○ 4 ○ 5

B.3 Etica e deontologia in TD e CPP*

○ 1 ○ 2 ○ 3 ○ 4 ○ 5

B.4 Criteri di eleggibilità per TD e CPP (ovvero le 4 categorie di patologia)*

○ 1 ○ 2 ○ 3 ○ 4 ○ 5

B.5 Modalità, criteri e metodi per la valutazione del dolore*

○ 1 ○ 2 ○ 3 ○ 4 ● 5

B.6 Percorsi assistenziali in TD e CPP e modalità di attivazione*

○ 1 ○ 2 ○ 3 ○ 4 ○ 5

B.7 Diritto del bambino/a ad essere informato/a*

○ 1 ○ 2 ○ 3 ○ 4 ○ 5

B.8 Modalità di comunicazione con il bambino/a (per età, sviluppo, consapevolezza, cultura, ...)*

○ 1 ○ 2 ○ 3 ○ 4 ○ 5

B.9 Modalità di comunicazione con i famigliari (afferenti a diversi contesti socio culturali)*

○ 1 ○ 2 ○ 3 ○ 4 ○ 5

B.10 Modalità per individuare i bisogni del bambino/a e della famiglia (emotivi, relazionali, spirituali, amicali, scolastici o lavorativi, ...) nel tempo della malattia*

○ 1 ○ 2 ○ 3 ○ 4 ○ 5

B.11 Bisogni clinici e assistenziali in TD e CPP*

○ 1 ○ 2 ○ 3 ○ 4 ○ 5

B.12 Interventi per il trattamento del dolore*

○ 1 ○ 2 ○ 3 ○ 4 ○ 5

B.13 Il concetto di qualità di vita in TD e CPP*

○ 1 ○ 2 ○ 3 ○ 4 ○ 5

B.14 Modalità per definire un'assistenza personalizzata nell'ottica della qualità di vita*

○ 1 ○ 2 ○ 3 ○ 4 ○ 5

B.15 Modalità di attivazione della continuità assistenziale tra ospedale e territorio*

○ 1 ○ 2 ○ 3 ○ 4 ○ 5

B.16 Modalità di transizione dai servizi pediatrici a quelli per l'età adulta*

○ 1 ○ 2 ○ 3 ○ 4 ○ 5

B.17 Bisogni psicologici e sociali di bambino/a e famiglia nel fine vita*

○ 1 ○ 2 ○ 3 ○ 4 ○ 5

B.18 Bisogni clinici, sintomi ed emergenze che si presentano nel fine vita*

1 2 3 4 5

B.19 Dilemmi etici nell'inguaribilità e nel fine vita*

1 2 3 4 5

B.20 Lutto*

1 2 3 4 5

C. QUANTO VORRESTI MIGLIORARE LA TUA CONOSCENZA SUI SEGUENTI TEMI (esprimi la tua valutazione su una scala da 1 a 5, dove 1=per nulla, 2=poco, 3=abbastanza, 4=molto e 5=moltissimo)

C.1 Valutazione di bambino e famiglia per l'accesso alle rete di CPP e TDP*

1 2 3 4 5

C.2 Accoglienza di bambino e famiglia nella rete di CPP e TDP*

1 2 3 4 5

C.3 Presa in carico di pazienti e famiglie nei diversi setting assistenziali della rete di CPP e TDP*

1 2 3 4 5

C.4 Lavoro in equipe*

1 2 3 4 5

C.5 Gestione del sè*

1 2 3 4 5

C.6 Attivazione e gestione di un centro di riferimento di CPP e TDP*

1 2 3 4 5

C.7 Formazione in CPP e TDP*

1 2 3 4 5

C.8 Ricerca in CPP e TDP*

1 2 3 4 5

D. QUAL E' LA RISPOSTA CORRETTA? (È possibile indicare solo una risposta per domanda)

D.1 Cosa comprende il quadro normativo che disciplina le CPP e TD? *

D.2 Quali sono le patologie del bambino eleggibili alla CPP?*

D.3 In quali casi il bambino ha diritto ad essere informato sulla sua prognosi?*

D.4 Il protocollo di Buckman è:*

D.5 Cosa è necessario per una presa in carico multi-professionale della famiglia del piccolo paziente?*

D.6 Quando muore un bambino siamo di fronte a:*

un lutto inelaborabile un lutto traumatico un lutto difficile un lutto normale

D.7 La differenza tra sedazione palliativa e sedazione terminale riguarda:*

D.8 L'attivazione della continuità assistenziale è subordinata a:*

D.9 Qual è il sintomo più frequente nel fine vita del bambino con patologia non oncologica?*

il dolore le difficoltà di alimentazione la perdita della motilità autonoma la fatica e i disturbi respiratori

Se hai piacere, indicaci i tuoi suggerimenti:

Conferma che non sei un bot risolvendo questa semplice operazione : **6 + 7** =

Invia le risposte

Table S2. Total scores by socio-demographic characteristics of respondents

Socio-demographic variables	Perceived Knowledge		Wished Knowledge		Actual Knowledge	
	Median (IQR)	p	Median (IQR)	p	Median (IQR)	p
**Gender**		0.803		0.789		0.467
Male, N=15	58.0 (46.0-52.0)		29.0 (24.0-39.0)		6.0 (4.0-7.0)	
Female, N=90	52.0 (43.0-66.0)		28.0 (24.0-34.0)		6.0 (5.0-7.0)	
**Age class**		0.833		0.642		0.357
≤ 30, N=12	50.0 (38.0-63.5)		32.0 (24.0-35.5)		7.0 (4.5-7.5)	
31-45, N=36	56.0 (43.5-66.0)		28.5 (25.0-37.5)		5.5 (4.0-7.0)	
46-60, N=49	50.0 (44.0-65.0)		28.0 (23.0-32.0)		6.0 (5.0-7.0)	
≥ 61, N=8	55.0 (46.0-67.5)		25.0 (23.5-32.0)		6.5 (5.5-7.0)	
**Amount of work with pediatric patients**		0.867		0.362		0.281
None, N=18	50.0 (38.0-68.0)		27.5 (22.0-32.0)		7.0 (6.0-7.0)	
< 50%, N=15	57.0 (46.0-71.0)		27.0 (24.0-31.0)		6.0 (5.0-7.0)	
> 50% and < 100%, N=17	54.0 (44.0-67.0)		27.0 (25.0-37.0)		6.0 (5.0-7.0)	
100%, N=55	52.0 (44.0-60.0)		31.0 (24.0-36.0)		6.0 (5.0-7.0)	
**Primary work setting**		0.234		***0.043***		0.676
Hospital and community, N=6	63.0 (54.0-85.0)		25.0 (23.0-28.0)		7.0 (6.0-8.0)	
Hospital, N=57	53.0 (44.0-64.0)		31.0 (25.0-38.0)		6.0 (5.0-7.0)	
Community, N=21	50.0 (45.0-70.0)		32.0 (27.0-33.0)		6.0 (5.0-7.0)	
Other, N=21	50.0 (60.0-40.0)		24.0 (23.0-29.0)		7.0 (4.0-7.0)	
**Length of service**		0.334		***0.027***		0.615
< 5 years, N=19	54.0 (47.0-67.0)		25.0 (16.5-28.5)		6.0 (4.0-7.0)	
5-10 years, N=12	46.5 (37.0-57.0)		30.0 (25.0-37.0)		4.5 (4.0-7.5)	
> 10 years, N=74	52.0 (44.0-66.0)		29.0 (24.0-35.0)		6.0 (5.0-7.0)	
**Prior education in PC/PPC**		***<0.001***		0.761		0.297
None, N=35	46.0 (36.0-55.0)		26.0 (22.0-37.0)		6.0 (4.0-7.0)	
Congresses/seminars, N=63	54.0 (47.0-67.0)		30.0 (25.0-35.0)		6.0 (5.0-7.0)	
Advanced courses in PC/PPC (with or without congresses/seminars), N=2	82.0 (65.0-84.0)		28.0 (24.0-29.0)		5.0 (5.0-6.0)	
Master in PC (with or without congresses/seminars/advanced courses), N=4	74.0 (53.0-88.0)		29.5 (22.0-33.0)		6.5 (5.5-7.5)	
**Prior work experience in PC/PPC**		***<0.001***		*0.085*		0.128
None, N=62	48.0 (41.0-55.0)		28.5 (23.0-33.0)		6.0 (4.0-7.0)	
Only PC, N=18	57.5 (40.0-66.0)		27.0 (23.0-30.0)		7.0 (5.0-8.0)	
Only PPC, N=15	59.0 (50.0-73.0)		34.0 (27.0-38.0)		7.0 (5.0-8.0)	
Both, N=10	75.0 (66.0-80.0)		28.5 (26.0-33.0)		6.0 (6.0-7.0)	

Abbreviations: PC = Palliative Care; PPC = Pediatric Palliative Care

Table S3. Proportion of scores ≥ 4 for items of Whished Knowledge scale by professional profile

**Wished Knowledge**	**Total N=102**	**Physicians N=15**	**Nurses N=56**	**Psychologists N=31**	**p**
Evaluation for PPC/PT network access, N (%)	63 (61.8)	17 (54.8)	36 (64.3)	10 (66.7)	0.627
Reception in the PPC/PT network, N (%)	59 (57.8)	15 (39.5)	36 (64.3)	8 (53.3)	0.330
Charge in the PPC/PT network settings, N (%)	60 (58.8)	17 (54.8)	35 (62.5)	8 (53.3)	0.704
Team work, N (%)	67 (65.7)	21 (67.7)	37 (66.1)	9 (60.0)	0.871
Management of self, N (%)	56 (54.9)	16 (51.6)	31 (55.4)	9 (60.0)	0.862
PPC/PT Reference center management, N (%)	51 (50.0)	11 (35.5)	32 (57.1)	8 (53.3)	0.148
Training on PPC/PT, N (%)	60 (58.8)	15 (39.5)	38 (67.9)	7 (46.7)	0.123
Research on PPC/PT, N (%)	43 (42.1)	7 (22.5)	31 (55.4)	5 (33.3)	***0.009***

Abbreviations: PC = Palliative Care; PPC = Pediatric Palliative Care
